# Ras signaling and RREB1 are required for the dissociation of medial edge epithelial cells in murine palatogenesis

**DOI:** 10.1242/dmm.049093

**Published:** 2022-02-15

**Authors:** Toshihiro Inubushi, Ayaka Fujiwara, Takumi Hirose, Gozo Aoyama, Toshihiro Uchihashi, Naoki Yoshida, Yuki Shiraishi, Yu Usami, Hiroshi Kurosaka, Satoru Toyosawa, Susumu Tanaka, Tetsuro Watabe, Mikihiko Kogo, Takashi Yamashiro

**Affiliations:** 1 Department of Orthodontics and Dentofacial Orthopedics, Osaka University Graduate School of Dentistry, Osaka 5650871, Japan; 2 First Department of Oral and Maxillofacial Surgery, Graduate School of Dentistry, Osaka University, Osaka 5650871, Japan; 3 Department of Oral Pathology, Graduate School of Dentistry, Osaka University, Osaka 5650871, Japan; 4 Department of Biochemistry, Graduate School of Medical and Dental Sciences, Tokyo Medical and Dental University, Tokyo 1138549, Japan

**Keywords:** Ras, RREB1, Medial edge epithelial cells, EMT, TGF-β3, Palatogenesis

## Abstract

Cleft palate is one of the major congenital craniofacial birth defects. The etiology underlying the pathogenesis of cleft palate has yet to be fully elucidated. Dissociation of the medial edge epithelium (MEE) at the contacting region of palatal shelves and subsequent migration or apoptosis of MEE cells is required for proper MEE removal. Ras-responsive element-binding protein 1 (RREB1), a RAS transcriptional effector, has recently been shown to play a crucial role in developmental epithelial–mesenchymal transition (EMT), in which loss of epithelial characteristics is an initial step, during mid-gastrulation of embryonic development. Interestingly, the involvement of RREB1 in cleft palate has been indicated in humans. Here, we demonstrated that pan-Ras inhibitor prevents the dissociation of MEE during murine palatal fusion. *Rreb1* is expressed in the palatal epithelium during palatal fusion, and knockdown of *Rreb1* in palatal organ culture resulted in palatal fusion defects by inhibiting the dissociation of MEE cells. Our present findings provide evidence that RREB1-mediated Ras signaling is required during palatal fusion. Aberrant RREB1-mediated Ras signaling might be involved in the pathogenesis of cleft palate.

## Abstract (precis)

**Summary:** RREB1, a known transcriptional factor that acts downstream of Ras signaling, is expressed in the medial edge epithelium (MEE) region and required for the dissociation of MEE during palatal fusion.

## INTRODUCTION

Cleft palate is one of the major congenital craniofacial birth defects that result from failure of growth, elevation, and fusion of the palatal shelves during embryogenesis (Dixon et al., 2011; Murray, 2002). This condition shows anatomical impairments combined with various defects involving the soft and hard palates, the nasal septum and the alveolar ridge. It is well accepted that cleft palate is a multifactorial disease caused by a combination of genes and environmental factors. Although recent studies using genetically modified mice have helped to identify the genetic and environmental etiology of cleft palate, the etiology underlying the pathogenesis of cleft palate has largely remained unknown.

In mice, palatal fusion begins at the midline of the palatal shelves following vertical palate elevation above the tongue and bilateral outgrowth of the palatal shelves toward each other. Following contact of the palatal shelves at the medial edge epithelium (MEE), the intervening epithelium between the palatal shelves merges to form the epithelium seam, which is subsequently removed to allow mesenchymal continuity across the fused palate (Bush and Jiang, 2012; Ferguson, 1988). Fusion of the palatal shelves is crucial for the correct formation of the palate, and its defect can lead to cleft palate.

Transforming growth factor-beta 3 (*Tgfb3*) has been established as a critical gene for causing cleft palate through regulating epithelial fusion in mice and humans (Lidral et al., 1998; Taya et al., 1999; Zhu et al., 2010). Although the roles of TGF-β3 have been widely studied in the dissociation of MEE during palatal fusion, the cross-talk between TGF-β3 and other signaling pathways in palatogenesis and the regulatory mechanism in that process remain unexplored (Nakajima et al., 2018).

The RAS proteins are members of a large superfamily of low-molecular-weight GTP-binding proteins. RAS plays pivotal roles in numerous basic cellular functions, including regulation of proliferation, differentiation and apoptosis (Downward, 2003). Physiological and oncogenic activation of RAS, encoded by one of three isoforms, *HRAS*, *KRAS* or *NRAS*, is coupled to a wide range of downstream signaling pathways, including RAF–MEK–ERK (RAF1–MAP2K–MAPK), phosphoinositide 3-kinases (PI3Ks) and Rho GTPases (Rajalingam et al., 2007).

Ras-responsive element-binding protein 1 (RREB1) is a zinc finger transcription factor that binds to RAS-responsive elements (RREs) of gene promoters in response to RAS signaling activation (Mukhopadhyay et al., 2007). Recently, Su et al. (2020) reported that RREB1 plays a pivotal role in developmental epithelial–mesenchymal transitions (EMTs) during mid-gastrulation of embryonic development by integrating TGF-β and Ras signals. Importantly, developmental EMTs are regulated by tissue-specific mechanisms and occur in a temporal and spatial manner. Studying the context-dependent regulatory molecular mechanisms of these developmental EMTs is very important for understanding cell plasticity during embryonic development and for clarifying the pathogenesis of developmental diseases, including cleft palate, in which EMT-like loss of epithelial characteristic is at least partially involved.

In humans, there is no direct genetic evidence for the involvement of *RREB1* mutation in palatal fusion defects. However, the association of *RREB1* with cleft palate has been indicated in some congenital disorders. Chromosome 6pter-p24 deletion syndrome (OMIM #612582) is a chromosomal disorder carrying a terminal deletion of the short arm of chromosome 6 (6p), the region that includes the *RREB1* gene. The 6pter-p24 deletion syndrome is characterized by developmental delay/mental retardation, reduced muscle tone, Dandy–Walker malformation, hearing loss, anomaly of eyes, cardiac abnormalities, and abnormal craniofacial features often associated with orofacial clefting (Davies et al., 2004; Topping et al., 2002). *RREB1* is also a candidate factor in the pathogenesis of DiGeorge syndrome, which is associated with craniofacial abnormalities, including cleft palate. The expression of *RREB1* is dysregulated by an epigenetic mechanism in DiGeorge syndrome. These observations indicate the involvement of the *RREB1* gene in the pathogenesis of cleft palate in humans. However, the role of RREB1 in the dissociation of MEE, a process with steps partially overlapping the biological process of developmental EMT, during palatogenesis has yet to be determined.

Here, we demonstrated the essential roles of Ras signaling in the dissociation of MEE during murine palatal fusion. In addition, the present study provides the first evidence that RREB1 is expressed at the fusing surface of the secondary palate and is required for the dissociation of MEE in palatogenesis.

## RESULTS

### Pan-Ras inhibitor prevents palatal fusion in organ culture

**Fig. 1.**
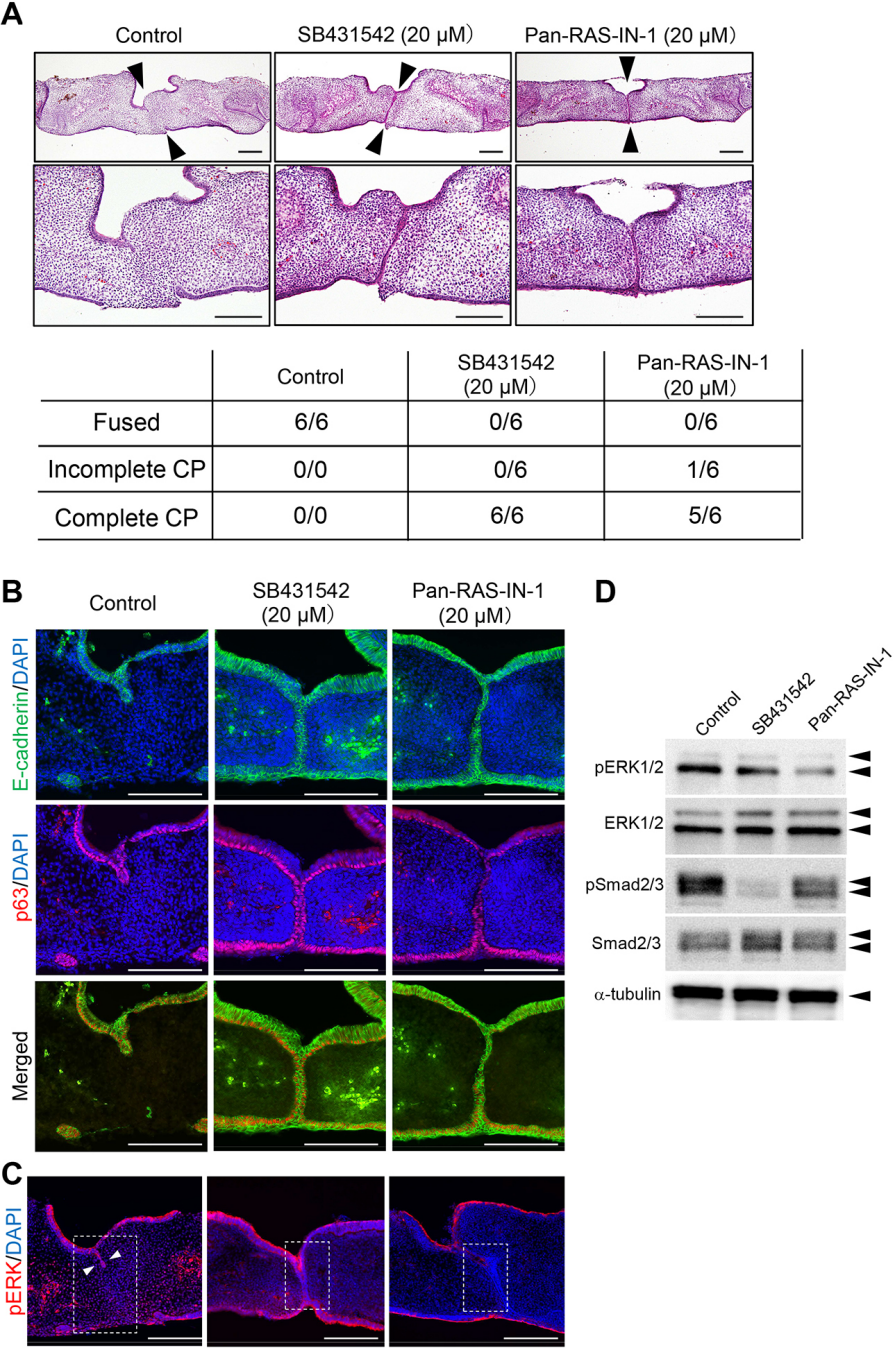
**Pan-Ras inhibitor prevents palate fusion in organ culture.** (A) Palatal shelf organ cultures were prepared using a pair of the dissected shelves from wild-type mice at E14.5, culturing for 48 h. Dissected shelves were pretreated with SB431542 (20 µM), Pan-Ras-IN-1 (20 µM) or dimethyl sulfoxide (DMSO) for 6 h prior to organ culture. Histological sections of horizontal palatal shelves showed the complete fusion defects of the palatal shelves and the remaining medial edge epithelium (MEE) in SB431542-treated and Pan-Ras-IN-1-treated groups. Arrowheads indicate the contact region of the paired shelves. The lower panel displays the incidence of palatal fusion in each treatment group. CP, cleft palate. (B) The persistent expression of E-cadherin and p63 was observed in the MEE region despite forced contact between the palatal shelves in the SB431542- and Pan-Ras-IN-1-treated groups. (C) The overall expression of phosphorylated (p)ERK is decreased in the Pan-Ras-IN-1-treated group. Note that positive expression was observed at the oral epithelial triangle region in the control culture (arrowheads). In contrast, the Pan-Ras-IN-1-treated cultures show substantially lower expression of pERK at the remaining MEE seam. (D) Western blotting confirmed the decreased expression of pERK in Pan-Ras-IN-1-treated cultures. The expression of pSmad2 is decreased by SB431542 treatment in cultured palatal shelves. Scale bars: 200 μm. The experiments in B, C and D were performed at least three times with similar results.

Palatal organ culture is a well-established method to examine the fusion of palatal epithelium (Brunet et al., 1995). To examine the roles of Ras signaling in palatal fusion, we used a pan-Ras inhibitor (Pan-Ras-IN-1) in palatal organ cultures. Pan-Ras-IN-1 has been well characterized as an inhibitor of Ras signaling (Welsch et al., 2017). We also used a specific TGF-β RI kinase (TGFBR1) inhibitor, SB431542, which has been demonstrated to inhibit palatal fusion in palatal organ cultures (Dudas et al., 2004; Inman et al., 2002). After 48 h of culture, control cultures showed complete fusion of the palatal shelves (Fig. 1A). The group treated with 20 μM SB431542 showed complete fusion defects of the palatal shelves covered with MEE cells. Similarly, treatment with 20 μM pan-Ras-IN inhibited palatal fusion in palatal organ cultures. E-cadherin is the most commonly used marker specific for the epithelial cells covering the palatal shelves. EMT-like loss of epithelial characteristics is an essential process in which E-cadherin disappears prior to the removal of the MEE during palatal fusion (Nawshad et al., 2004). p63 (TP63) is one of the key transcriptional factors for which mutation results in cleft palate in humans and mice (Thomason et al., 2008; van Bokhoven and Brunner, 2002). Downregulation of p63 in MEE of the palatal shelves is required for palatal fusion by promoting periderm migration and reducing the proliferative potential of MEE (Richardson et al., 2017). The immunostaining of E-cadherin and p63 demonstrated that E-cadherin and p63 double-positive MEE cells disappeared from the contact region of the palatal shelves after 2 days of culture in the control group (Fig. 1B). In contrast, persistent expression of E-cadherin and p63 was observed in the MEE, despite forced contact between the palatal shelves in the SB431542- and Pan-Ras-IN-1-treated groups. Because the ERK pathway is one of the downstream pathways of Ras proteins, we examined the effects of pan-Ras-IN on the expression of phosphorylated (p)ERK in the organ cultures. Pan-Ras-IN-1-treated cultures showed substantially lower expression of pERK at the remaining MEE seam, as shown by immunohistochemical staining (Fig. 1C). Interestingly, Yamamoto et al. demonstrated that EGF treatment of palatal explants prevented palatal fusion and MEK inhibitor rescued the EGF-induced inhibition of palatal fusion (Yamamoto et al., 2003). These observations indicate that appropriate activation of the ERK pathway is required for the normal process of palatal fusion. Western blotting demonstrated decreased expression of pERK in Pan-Ras-IN-1-treated cultures and pSmad2/3 in SB431542-treated cultures (Fig. 1D). Taken together, our results indicated that Ras signaling is required for the dissociation of the MEE, which is an essential process in palatal fusion.

### Pan-Ras inhibitor attenuates MEE cell migration in unpaired palatal explants

**Fig. 2.**
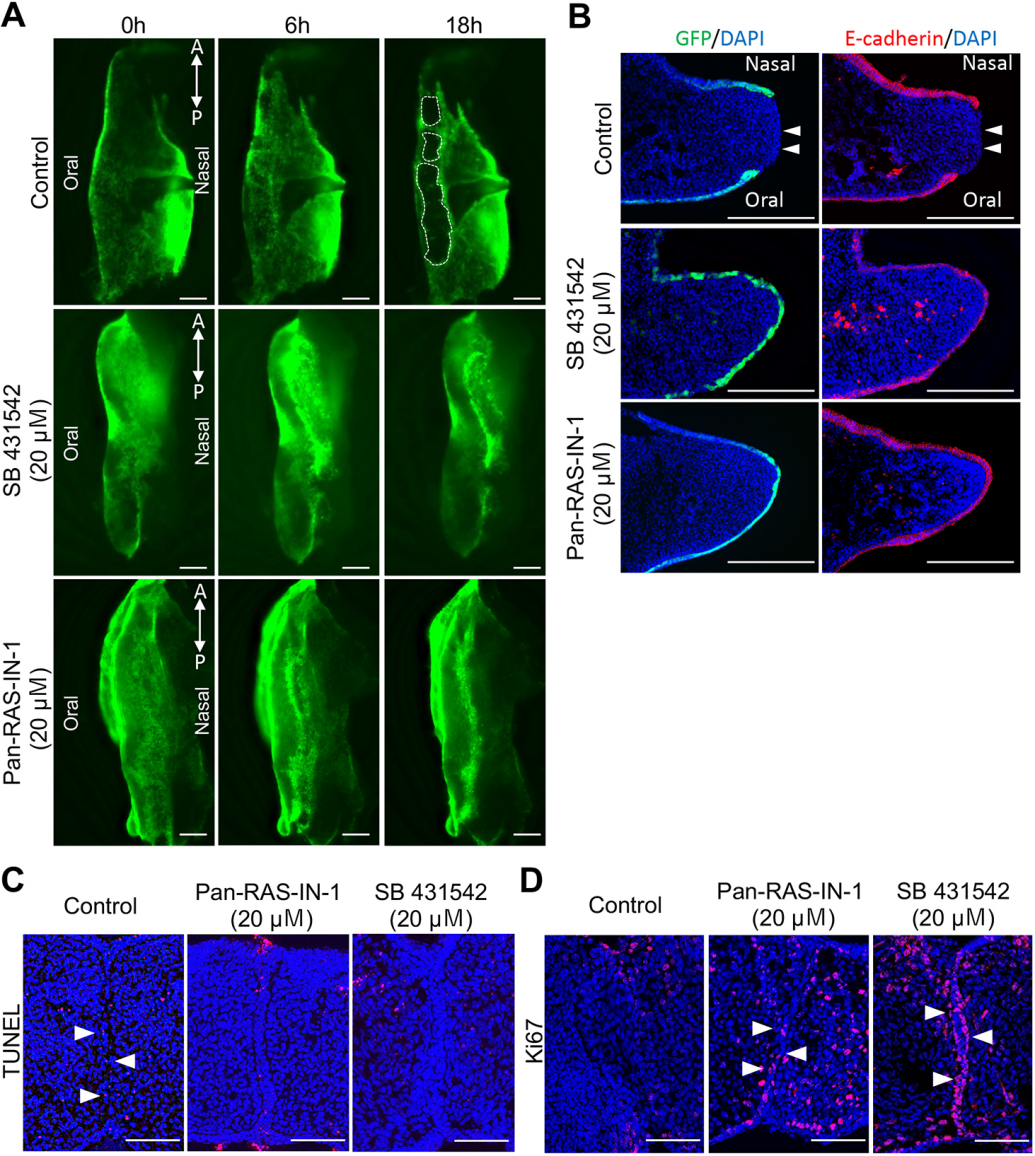
**Pan-Ras inhibitor attenuates MEE cell migration and apoptosis in unpaired palatal explants.** Epithelial behavior in the unpaired palatal explant model. (A) Fluorescence microscopic images showing the medial edges of the unpaired palatal explant model after 0, 6 and 18 h of culture. White dashed lines indicate the area of mesenchymal exposure. SB431542 (20 µM) and Pan-Ras-IN (20 µM) treatment completely inhibited the migration of MEE cells and subsequent mesenchymal exposure. (B) Frontal section of the palatal shelf of K14-GFP mice after 18 h of unpaired culture. E-cadherin expression is retained at the MEE region in both the SB431542-treated and Pan-Ras-IN-treated groups. Arrowheads indicate the mesenchyme exposure site. (C) Analysis of cell death after 24 h of palatal shelf organ cultures. The number of TUNEL-positive cells in remaining MEE cells (arrowheads) is markedly reduced in the Pan-Ras-IN-treated and SB431542-treated groups. (D) Analysis of cell proliferation after 24 h of palatal shelf organ culture. Proliferative activity is maintained in the remaining MEE cells (arrowheads) in the Pan-Ras-IN-treated and SB431542-treated groups. A, anterior; P, posterior. Scale bars: 200 μm (A,B); 100 μm (C,D). These experiments were performed at least three times with similar results.

We previously established an unpaired palatal explant model using epithelial-GFP-labeled mice for tracing the behavior of MEE cells at the developing secondary palate (Aoyama et al., 2019). In this unpaired palatal explant culture system, MEE cells can disappear from the medial edge of the single palatal shelf independently from palatal shelf contact and midline seam formation. Furthermore, the distribution and timing of this epithelial removal are closely similar to those of the disappearance of the MEE reported *in vivo* in previous studies (Charoenchaikorn et al., 2009; Takigawa and Shiota, 2007). Although the unpaired palatal explant culture system has some limitations, this culture system enables us to monitor epithelial behavior, including epithelial cell migration on the surface of the secondary palatal shelf. Therefore, we used the unpaired palatal explant model to further examine the role of Ras signaling in the cellular behavior of MEE cells during palatal fusion. The results showed that MEE cell migration and subsequent mesenchymal exposure were strongly disturbed in both the 20 μM SB431542-treated and 20 μM pan-Ras-IN-treated groups (Fig. 2A). In addition, frontal section confirmed that the palatal shelves were covered with the MEE cells, and E-cadherin expression was retained at the MEE region, in both the SB431542-treated and pan-Ras-IN-treated groups (Fig. 2B). These results suggest that Ras signaling is required for the loss of E-cadherin that is an essential process of MEE dissociation in palatogenesis.

### Pan-Ras inhibitor reduces apoptosis of MEE cells in organ culture

Apoptosis is also an important cellular event during MEE removal from the fusing palatal shelf. Next, we examined the effects of pan-Ras inhibition on the apoptosis of MEE cells in palatal organ culture. Terminal deoxynucleotidyl transferase dUTP nick end labeling (TUNEL) assays revealed that the number of TUNEL-positive cells in the remaining MEE cells was markedly reduced in the pan-Ras-IN-treated and SB431542-treated groups compared to the control group (Fig. 2C). Ki67 (Mki67) immunostaining demonstrated that the proliferation activity was maintained in the remaining MEE cells in the pan-Ras-IN-treated and SB431542-treated groups (Fig. 2D). Together, these results demonstrated that Pan-Ras inhibitor-treated MEE cells continued to proliferate and did not undergo cell death, and these observations are consistent with the effects of TGF-β RI kinase inhibitor application.

### Synergistic effects of TGF-β RI kinase inhibitor and pan-Ras inhibitor during palatal fusion

**Fig. 3.**
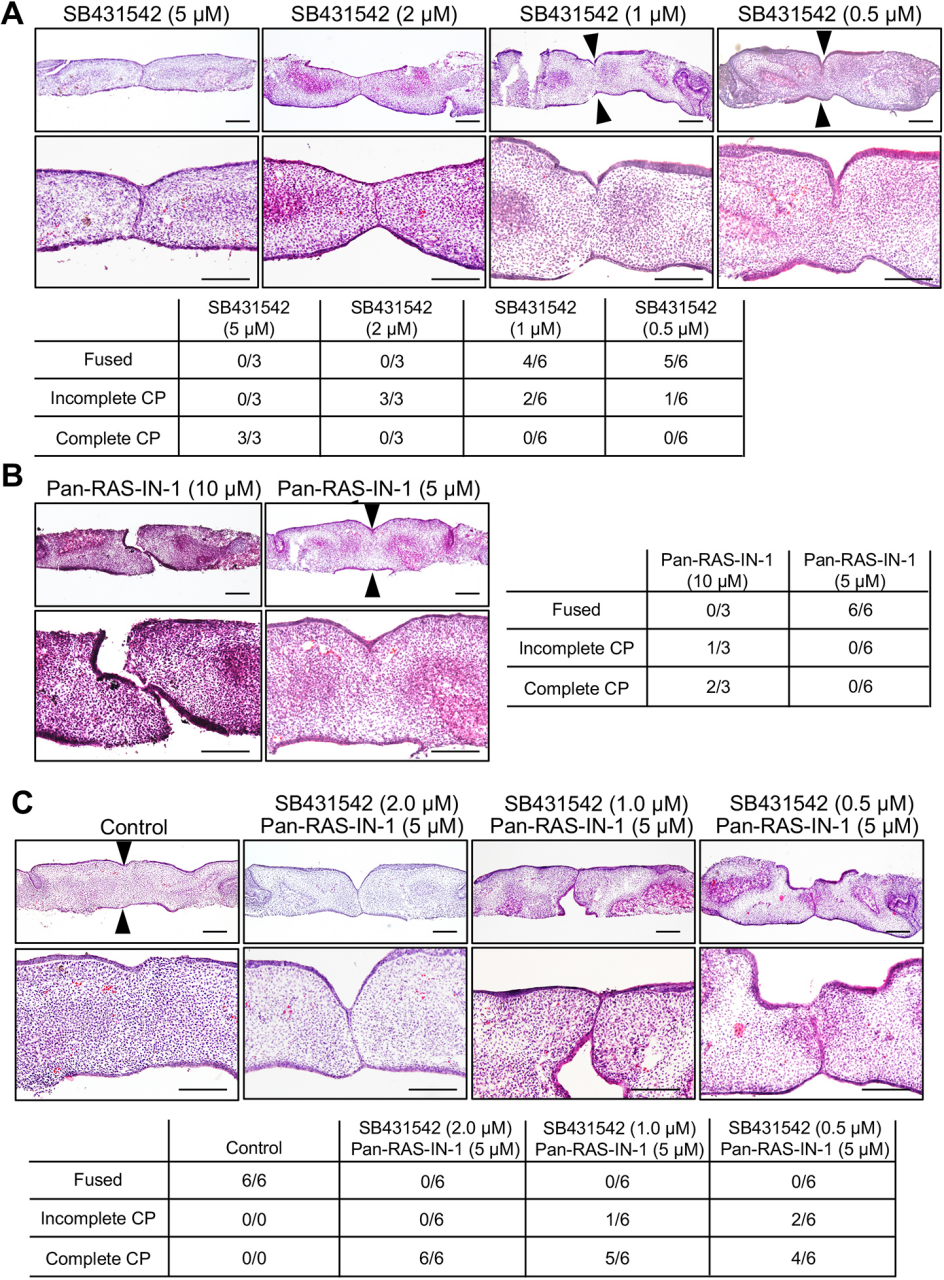
**Synergistic effects of TGF-β RI kinase inhibitor and pan-Ras inhibitor during palatal fusion.** Palatal shelf organ cultures were prepared using a pair of the dissected shelves from wild-type mice at E14.5, culturing for 48 h with specific inhibitor or DMSO as described below. (A) Dissected shelves were pretreated with 5, 2, 1 or 0.5 µM SB431542 or DMSO for 6 h prior to organ culture. (B) Dissected shelves were pretreated with 10 µM or 5 µM Pan-Ras-IN-1 or DMSO for 6 h prior to organ culture. (C) Simultaneous treatments with SB431542 and Pan-Ras-IN-1 at the indicated concentrations were performed 6 h prior to organ culture. Histological sections of horizontal palatal shelves were stained with Hematoxylin and Eosin (HE). Arrowheads indicate the contact region of the paired shelves in fused palatal shelves. The lower (right in B) panel displays the incidence of palatal fusion in each treatment group. Scale bars: 200 μm.

We also conducted simultaneous application of SB431542 and Pan-Ras-IN-1 to examine the synergistic effects of TGF-β3 signaling and Ras signaling on palatal fusion in palatal organ culture. A single application of SB431542 at lower than 1 μM concentration or Pan-Ras-IN-1 at lower than 5 μM concentration failed to inhibit the fusion of palatal shelves (Fig. 3A,B). However, simultaneous application of 0.5 μM SB431542 and 5 μM Pan-Ras-IN-1 attenuated palatal fusion by blocking the removal of MEE in palatal organ culture (Fig. 3C). These synergistic effects of SB431542 and Pan-Ras-IN-1 suggest a cooperative role of TGF-β3 signaling and Ras signaling in the dissociation of MEE during palatal fusion.

### *Rreb1* is expressed in the palatal epithelium during palatogenesis

**Fig. 4.**
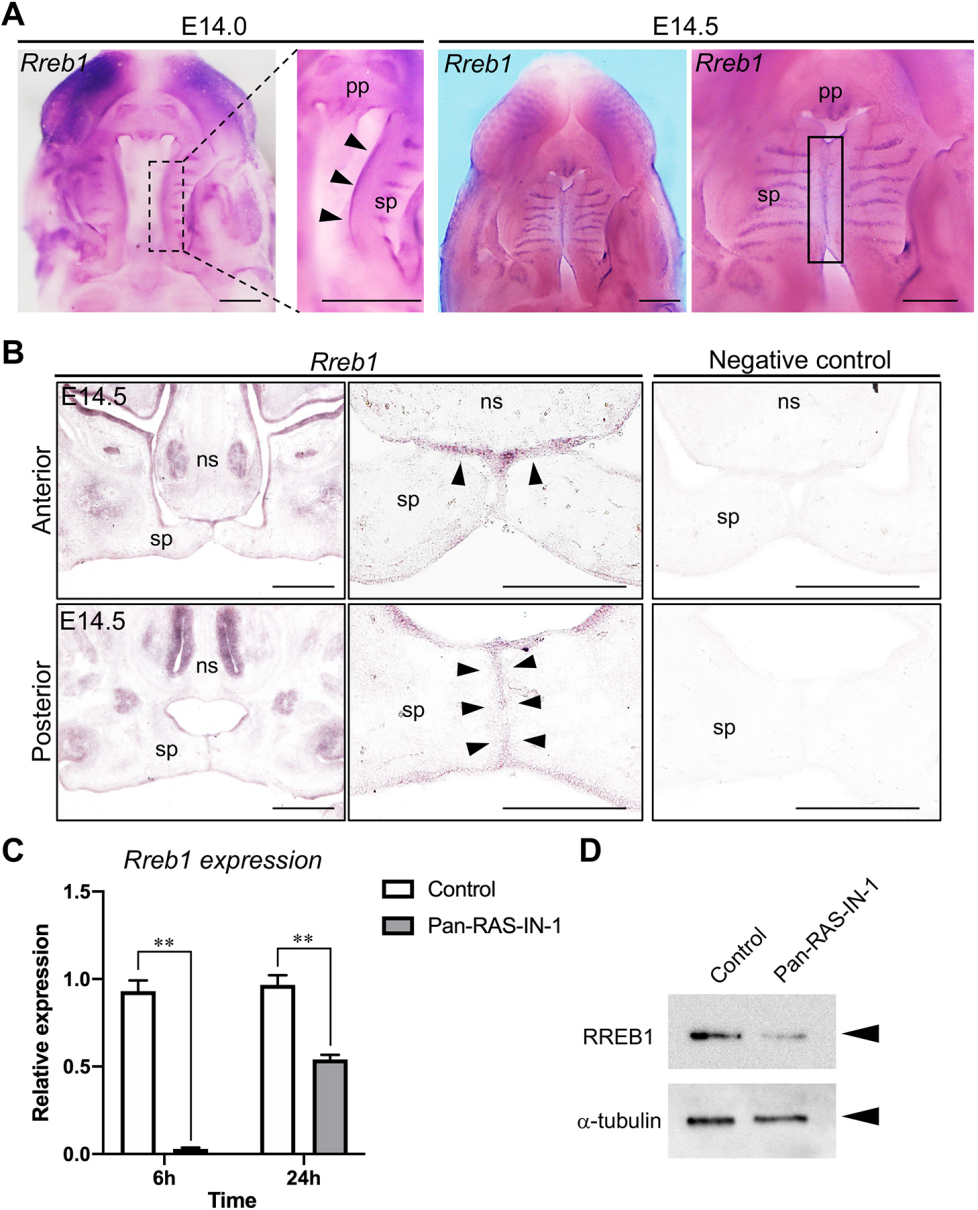
***Rreb1* expression in the palatal epithelium during palatogenesis.** (A) Whole-mount *in situ* hybridization of *Rreb1* in the developing palate of the wild type at E14.0 and E14.5. *Rreb1* was widely distributed in the MEE region of the secondary palate along the anterior–posterior axis and palatal rugae at E14.0. The expression of *Rreb1* was also prominent in the midline region of the fusing secondary palate. Arrowheads indicate the MEE region of the secondary palate. Box denotes the MEE region of the fusing palatal shelves. (B) Frontal section of the anterior and posterior region of palatal shelves in wild type. *Rreb1* was specifically expressed in oral epithelium, including fusing MEE at the anterior and posterior region. Arrowheads indicate the expression of *Rreb1* in fusing MEE. (C) Expression of *Rreb1* in palatal shelf organ cultures with or without 20 µM Pan-Ras-IN-1. *Rreb1* expression was significantly decreased after 6 h and 24 h of Pan-Ras-IN-1 application. The expression of *Rreb1* was evaluated by qPCR. *Gapdh* was used as an internal control for normalization. Means±s.d. (*n*=3) are shown as horizontal bars. **P<0.01 (two-way ANOVA). (D) Palatal shelves from organ cultures with or without 20 µM Pan-Ras-IN-1 for 24 h were lysed in RIPA buffer. Cell lysates were immunoblotted with antibodies against RREB1 and α-tubulin. ns, nasal septum; pp, primary palate; sp, secondary palate. Scale bars: 500 μm.

RREB1 has recently been shown to play a crucial role in developmental EMT during mid-gastrulation of embryonic development (Su et al., 2020). We hypothesized that RREB1 might be involved in the dissociation of MEE during palatal fusion. However, the expression pattern and contribution of *Rreb1* in palatogenesis have yet to be examined. To this end, we examined the expression patterns of *Rreb1* mRNA in palatogenesis. In whole-mount preparations at embryonic day (E)14.0 and E14.5, *Rreb1* mRNA was expressed at the MEE region of the secondary palate and palatal rugae (Fig. 4A). The expression of *Rreb1* was also prominent at the fusing surface of the secondary palate (Fig. 4B). These observations suggest that *Rreb1* may play an essential role in the epithelium, including MEE, rather than the mesenchyme, of palatal shelves in palatogenesis.

### Pan-Ras inhibitor downregulates the expression of *Rreb1* in palatal organ culture

Next, we examined the effects of Ras signaling inhibition on the expression of *Rreb1* in the palatal shelf during palatal fusion*.* Pan-Ras-IN treatment dramatically decreased the expression of *Rreb1* after 6 h of inhibitor application (Fig. 4C). Even after 24 h of inhibitor application, *Rreb1* expression was still downregulated compared to the level in the control palatal shelf (Fig. 4C). Consistently, Pan-Ras-IN treatment for 24 h decreased RREB1 protein expression in the palatal shelf (Fig. 4D). These results indicate that Ras signaling is essential for the expression of *Rreb1* in palatal shelves.

### Knockdown of *Rreb1* prevents palate fusion in organ culture

**Fig. 5.**
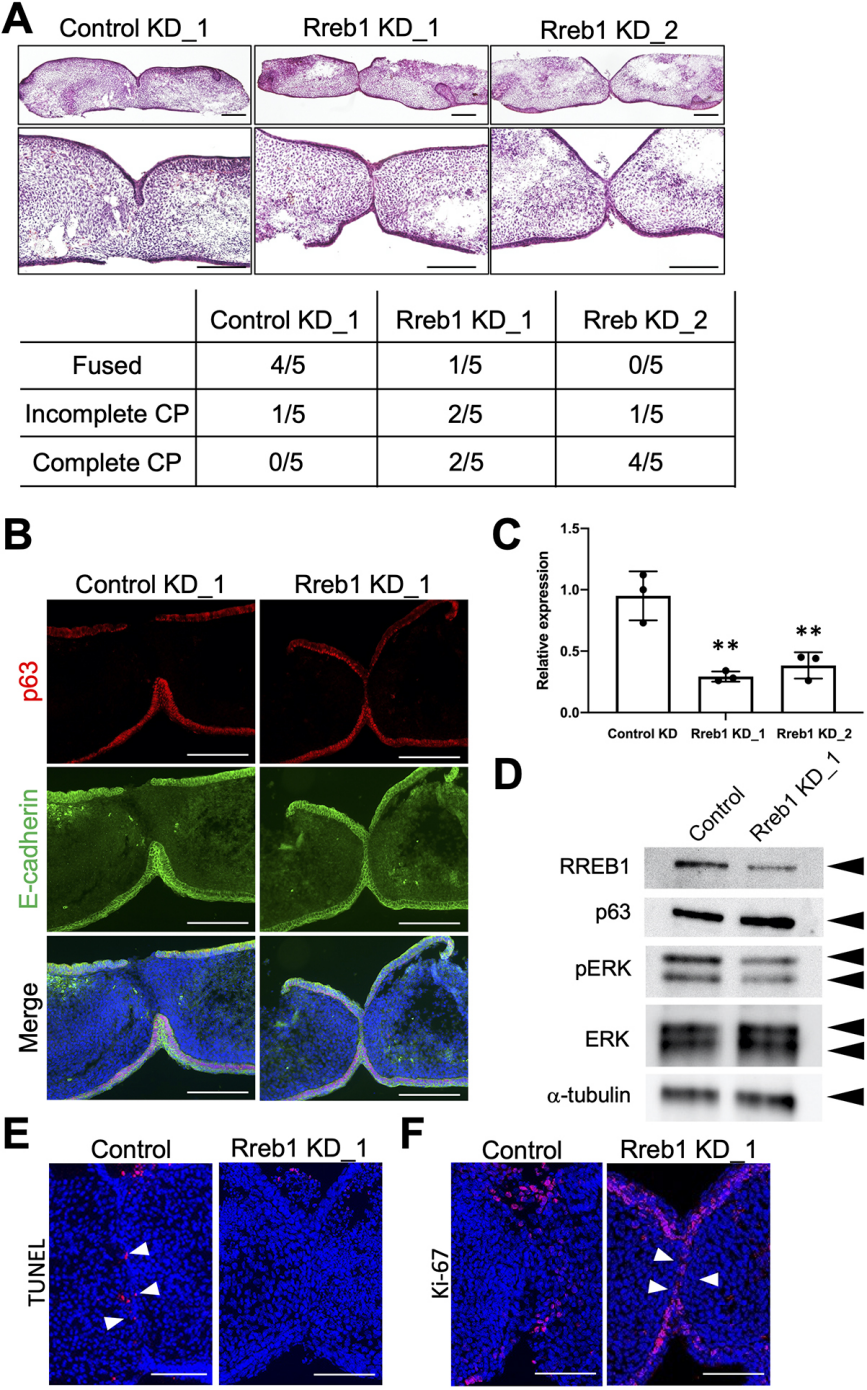
**Knockdown of *Rreb1* prevents palatal fusion in organ culture.** (A) Knockdown of *Rreb1* by siRNA was performed on dissected shelves 48 h prior to the paired palatal shelf organ culture. Then, the paired palatal shelf was cultured for 24 h. Histological sections of horizontal palatal shelves showed the fusion defects of the palatal shelves and the remaining MEE in the *Rreb1* knockdown group. Histological sections of horizontal palatal shelves were stained with HE. The incidence of fusion of palatal shelves in each treatment group is shown in the lower panel. (B) Persistent expression of E-cadherin and p63 was observed at the contacting palatal shelves in the *Rreb1* knockdown group. (C) Knockdown of *Rreb1* by siRNA was performed on dissected shelves for 48 h. RNA was extracted from palatal shelves in each group. The expression of *Rreb1* was evaluated by qPCR. *Gapdh* was used as an internal control for normalization. Means±s.d. (*n*=3) are shown as horizontal bars. **P<0.01 (two-way ANOVA). (D) Palatal shelves from control culture and *Rreb1* knockdown culture were lysed in RIPA buffer. Cell lysates were immunoblotted with antibodies against RREB1, p63, pERK, ERK and α-tubulin. This experiment was performed three times with similar results. (E,F) Analysis of cell death and cell proliferation in palatal shelf organ cultures. Control and *Rreb1* knockdown palatal shelves were cultured for 24 h. (E) The number of TUNEL-positive cells in remaining MEE cells (arrowheads) is markedly reduced in *Rreb1* knockdown cultures. (F) The proliferation activity is maintained in the remaining MEE cells (arrowheads) in *Rreb1* knockdown cultures. Scale bars: 200 μm (A,B); 100 μm (E,F).

RNA interference (RNAi) has been utilized within a palate organ culture model to specifically and efficiently knock down the target gene with low toxicity. To examine the potential roles of *Rreb1* in MEE during palatal fusion, we used siRNA to knock down *Rreb1* in palatal shelf organ culture experiments. *Rreb1* knockdown cultures showed incomplete fusion of the palatal shelves (Fig. 5A). Expression of E-cadherin and p63 was retained in the MEE seam of *Rreb1* knockdown cultures, as detected by immunohistochemical staining (Fig. 5B). TUNEL staining demonstrated that the number of TUNEL-positive cells in the remaining MEE cells was substantially decreased in *Rreb1*-knockdown cultures compared to the control cultures. Ki67 immunostaining revealed that the proliferative activity was retained in the remaining MEE cells in *Rreb1* knockdown cultures (Fig. 5E,F). These effects of *Rreb1* knockdown in palate organ cultures were consistent with the effects observed in pan-Ras inhibitor-treated palate organ cultures.

The decreased expression of *Rreb1* mRNA and RREB1 protein in *Rreb1* knockdown palatal shelves was confirmed by quantitative PCR (qPCR) and western blotting, respectively (Fig. 5C,D). The expression of pERK was slightly decreased in the *Rreb1* knockdown cultures (Fig. 5D).

## DISCUSSION

**Fig. 6.**
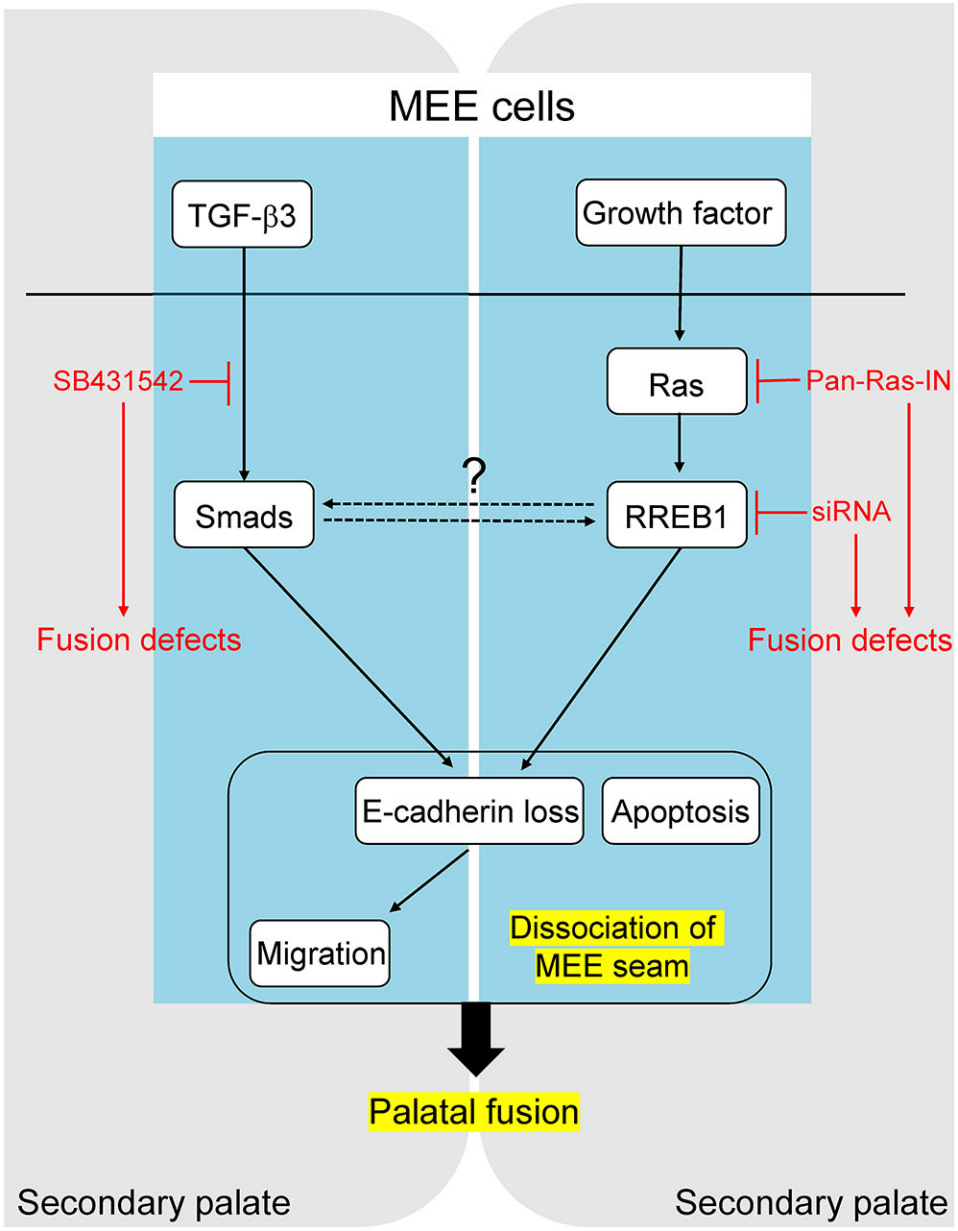
**Schematic of the key findings.** Ras signaling and its effector RREB1 are required for the dissociation of MEE seam in palatogenesis.

For the cellular mechanisms underlying MEE removal in palatal fusion, MEE seam degradation and subsequent MEE migration has been proposed as the major model. TGF-β isoforms have been extensively examined as the central signaling molecules in cancer and developmental EMT. However, it has been shown that the activation of TGF-β signaling is insufficient for complete EMT (Safina et al., 2009). RAS signaling is also well known to modulate the EMT process, particularly in cancer (Rajalingam et al., 2007). Interestingly, the cooperative roles of RAS and TGF-β–SMAD signaling in EMT have been reported previously in human cancer cells and human keratinocyte HaCaT cells. p63 is a critical regulator of epithelial cell proliferation and differentiation and acts downstream of TGF-β–SMAD and RAS signaling. It was reported that the ΔNp63 isoform, having the opposite function to p63, is necessary for the activation of the RAS signaling-dependent EMT gene program induced by TGF-β in multiple breast cancer cells (Sundqvist et al., 2020). Although the role of EMT in palatal fusion remains controversial, the dissociation of MEE at least partly shares morphological and molecular mechanisms with EMT. In palatogenesis, TGF-β3-induced downregulation of p63 in the MEE is required for palatal fusion by promoting MEE migration and reducing the proliferative potential of the MEE seam (Richardson et al., 2017). Note that downregulation of p63 in *Tgfb3*^−/−^ mice can restore MEE cell fate. In the present study, we demonstrated that Ras signaling, via its effector RREB1, regulates the dissociation of MEE and the subsequent migration of MEE cells during palatogenesis (Fig. 6). In this context, the recent indication of TGF-β signaling and Ras signaling cross-talk through the interaction between Smad protein and RREB1 in the process of palatal fusion is of particular interest.

Ras signaling mediates receptor tyrosine kinase (RTK) signaling, including FGF, PDGF and EGF signaling. FGFR1 and FGFR2 knockout mouse models demonstrated the crucial role of FGF signaling for cell proliferation in the growth of palatal shelves (Hosokawa et al., 2009; Kawai et al., 2019). PDGFRA mutant mice developed cleft palate due to a defect of the palatal shelf growth associated with the reduced extracellular matrix production. Unlike FGFR and PDGFR signaling, which is essential for the growth of palatal mesenchyme, EGFR signaling is primarily crucial in the palatal epithelium during palatal fusion. Although the penetration of the cleft palate phenotype is not high, EGFR knockout mice show cleft palate due to defects in MEE seam disruption (Miettinen et al., 1999). In addition, the involvement of EGFR signaling in EMT has been reported in cancer progression and metastasis (Lo et al., 2007). Thus, EGFR might be a major candidate receptor involved in the activation process of Ras–RREB1 signaling during palatal fusion.

Although cleft palate phenotype is not a major clinical feature in RASopathies, there are some indications regarding the association of RAS signaling with the pathogenesis of cleft palate in humans. Couser et al. reported that some cases exhibited cleft lip and/or palate phenotype in Noonan-like syndrome (Couser et al., 2018). High and narrow palate with submucous clefting is a typical craniofacial feature in cardiofaciocutaneous syndrome, which is caused by mutations in *KRAS*, *BRAF*, *MAP2K1* and *MAP2K2* (Chrzanowska et al., 1989). In addition, microdeletions within chromosome 22q11.2 exhibit haplo-insufficient ERK2 expression and reportedly cause cleft palate (Ben-Shachar et al., 2008).

A limitation of the present study is our lack of use of a genetically engineered mouse with *Rreb1* defect for the analysis of palatal fusion. The conventional *Rreb1* knockout mice exhibit lethality at early embryonic development (Morgani et al., 2021; Su et al., 2020). Thus, we cannot examine the cleft palate phenotype in *Rreb1* knockout mice using the conventional knockout mouse model. Although conditional knockout mice targeting *Rreb1* using the Cre/loxP system have not been generated yet, we may be able to test the phenotype of epithelial-specific knockout of *Rreb1* in the future. Although our approaches using small-molecule inhibitors and RNAi in palate organ cultures cannot completely rule out non-specific off-target effects, these approaches have been accepted as an alternative method when a genetic mouse model is not available. Another limitation of the study is the use of MEE cells for *in vitro* cell biological experiments*.* To the best of our knowledge, there are no available MEE cells or normal oral epithelial cells for analyzing the molecular mechanism of MEE dissociation in palatogenesis. Establishing MEE cells that can mimic MEE dissociation in palatal fusion would be an excellent approach to examine the molecular interactions and signaling cross-talk in palatogenesis.

In conclusion, the present study has demonstrated that Ras signaling is essential for dissociation of MEE during palatogenesis. RREB1, a known transcriptional factor that acts downstream of Ras signaling, is expressed in the MEE region and required for the dissociation of MEE during palatal fusion. With these findings, we provide essential information on the requirement for the Ras–RREB1 signaling axis in the dissociation of MEE in palatogenesis. However, the upstream activators of Ras signaling and the downstream molecules that activate RREB1 during palatal fusion remain to be elucidated.

## MATERIALS AND METHODS

### Animals

We used transgenic mice in which GFP was expressed under the control of the cytokeratin-14 promoter [K14 (KRT14)-GFP] (Vaezi et al., 2002). Mice expressing the transgene were identified by the green fluorescent glow of the skin surface. Mature female C57BL/6J mice (CLEA, Tokyo, Japan) were mated overnight with a K14-GFP male mouse, and the day on which a vaginal plug was found was designated as day 0 of pregnancy. Time-course observation of palatal shelf development was performed by dissecting K14-GFP mouse maxilla from E14.0 to E15.5.

### Dissection and organ culture

On E14, wild-type C57BL/6J mouse embryos were quickly immersed in BGJb medium (Gibco). The palatal shelves were removed using forceps under a dissecting microscope. Isolated palatal shelves were placed in pairs on 0.4 μm porosity filters (Millipore, Burlington, MA, USA), nasal epithelium down, media edges in contact, on 35 mm tissue culture dishes (FALCON, France). The culture medium was composed of BGJb medium with or without Pan-Ras-IN-1 and/or SB431542. Pan-Ras-IN-1 was purchased from MedChemExpress (Monmouth Junction, NJ, USA). SB431542 was purchased from Sigma-Aldrich (St Louis, MO, USA). Samples were pretreated for 6 h prior to organ culture. Palatal shelves were cultured at 37°C with 5% CO_2_. The culture medium and treatment solutions were replaced every 24 h.

### Time-lapse imaging and quantitative analysis

Time-lapse images of explant cultures were captured using an all-in-one fluorescence microscope (BZ-X700, Keyence, Osaka, Japan), equipped with filters for GFP (excitation, 475 nm; emission, 525 nm) and 4′,6-diamidino-2-phenylindole (DAPI; excitation, 360 nm; emission, 460 nm) channels. The instrument was controlled by the BZ Viewer version 1.0 software of the microscope (Keyence).

### Ethics

All animal experiments were performed in strict accordance with the guidelines of the Animal Care and Use Committee of the Osaka University Graduate School of Dentistry, Osaka, Japan. The protocol was approved by the Committee on the Ethics of Animal Experiments of Osaka University Graduate School of Dentistry. Mice were housed in the animal facility at the Department of Dentistry, Osaka University. Welfare guidelines and procedures were performed with the approval of the Osaka University Graduate School of Dentistry Animal Committee.

### Assessment of palatal fusion and histological analysis

The palatal phenotypes were first evaluated with a dissecting microscope. For histology, dissected samples were fixed in 4% paraformaldehyde, equilibrated in graded sucrose and embedded in Tissue-Tek (OCT compound, Sakura). The tissue samples were sectioned into 15 μm slices.

### Immunohistochemistry and TUNEL staining

Immunostaining of frozen sections was performed as previously described (Inubushi et al., 2017). The following antibodies were used: polyclonal rabbit anti-E-cadherin (1:50; ab15148, Abcam), monoclonal rabbit anti-Ki67 (1:50; ab16667, Abcam), monoclonal mouse anti-p63 (1:50; B1320, Santa Cruz Biotechnology), Alexa Fluor 488-labeled goat anti-rabbit IgG (1:500; A11034, Invitrogen), Alexa Fluor 555-labeled goat anti-mouse IgG (1:500; A21422, Invitrogen) and Alexa Fluor 555-labeled goat anti-rabbit IgG (1:500; A21428, Invitrogen). Apoptotic cells were identified using an *in situ* cell death detection kit (11684795910, Roche), according to the manufacturer's instructions.

### Immunoblotting

Protocols for immunoblotting were as described previously (Inubushi et al., 2018). Briefly, cells were lysed in ice-cold RIPA buffer containing 50 mmol/l Tris-HCl buffer (pH 7.6), 150 mmol/l NaCl, 1% Nonidet P40 Substitute, 0.5% sodium deoxycholate and 0.1% SDS. Protease inhibitor cocktail was purchased from Promega (Walldorf, Germany). Following a 30 min lysis period on ice, lysis samples were centrifuged at ∼20,000 ***g*** for 20 min at 4°C to prepare cell lysates. Then, 10 µg of lysate was subjected to SDS-PAGE on an 8-16% Tris-glycine gel (Invitrogen), followed by electroblotting onto an Immobilon PVDF membrane (EMD Millipore). ECL Western Blotting Substrate (07880, Nacalai Tesque) was used to detect signals. The following antibodies were used: monoclonal mouse anti-RREB1 (1:200; B-7, Santa Cruz Biotechnology), monoclonal rabbit anti-pSmad2/3 (1:1000; 8685, Cell Signaling Technology), monoclonal rabbit anti-Smad2/3 (1:1000; 8685, Cell Signaling Technology), monoclonal rabbit anti-pERK (1:1000; 4370, Cell Signaling Technology), polyclonal rabbit anti-ERK (1:1000; 9102, Cell Signaling Technology), α-tubulin (1:1000; T6074, Sigma-Aldrich), horseradish peroxidase-conjugated goat anti-rabbit IgG (1:500; 1706565, Bio-Rad) and horseradish peroxidase-conjugated goat anti-mouse IgG (1:500; 1706515, Bio-Rad).

### siRNA transfection in palatal culture system

E13.5 palatal shelves were pre-cultured in serum- and antibiotic-free medium for 4 h. The medium was then replaced with siRNA-containing medium according to the manufacturer's instructions. Transfection Reagent (Lipofectamine RNAiMax, Invitrogen) was mixed with siRNA targeting mouse *Rreb1* or scrambled control (Silencer Select siRNAs, Thermo Fisher Scientific). We used two different siRNAs for mouse *Rreb1*. Cultured palatal tissues were harvested after 48 h for organ culture, immunoblotting or real-time PCR procedures.

### RNA extraction and qPCR analysis

The E14.0 palatal shelves were incubated with or without Ras inhibitor for 6 h and 24 h. After the cultures, we dissected only the MEE region as much as possible using forceps under a dissecting microscope. Protocols for RNA extraction and qPCR analysis were as described previously (Inubushi et al., 2012). Total RNA was extracted from the dissected tissues using IsogenII (Nippon Gene) according to the manufacturer's protocol, then reverse transcribed to cDNA using oligo (dT) with reverse transcriptase (Takara). For real-time PCR, aliquots of total cDNA were amplified with TaqMan Fast Universal PCR Master Mix (Applied Biosystems, Foster City, CA, USA). Data acquisition and analysis were performed with a Step One Real-Time PCR System using Step One Software, Version 2.1 (Applied Biosystems). The PCR products were quantified using *Gapdh* as the reference gene. The primers and TaqMan probes were purchased from Applied Biosystems.

### Whole-mount *in situ* hybridization

Whole-mount *in situ* hybridization was performed using fixed E14.0, E14.5 and E15.0 palates. The digoxigenin (DIG)-labeled RNA probes were prepared using a DIG RNA-labeling kit according to the manufacturer's protocol (Roche), employing each cDNA clone as the template. The probes were synthesized from fragments of *Rreb1* (Allen Institute for Brain Science; https://alleninstitute.org/) and amplified with T7 and SP6 adaptor primers through PCR, as described previously (Sarper et al., 2018). After hybridization, the signals were visualized according to their immunoreactivity with anti-DIG alkaline phosphatase-conjugated Fab fragments (Roche).

### Statistics

Statistical methods were not used to predetermine sample size. Statistical analyses were performed with GraphPad Prism 8. Student's two-tailed *t*-test and two-way ANOVA were used under the assumption of normal distribution and observance of similar variance. *P*<0.05 was considered significant. Bonferroni post hoc analysis was performed where applicable. Values are expressed as mean±s.d. For all of these experiments, variances between groups were similar and data were symmetrically distributed. Data shown are representative images; each analysis was performed on at least three mice per genotype. Immunostaining was performed at least in triplicate. For other experiments, the numbers of biological replicates, animals or cells are indicated in the text.
